# Challenges Facing CRISPR/Cas9-Based Genome Editing in Plants

**DOI:** 10.3389/fpls.2022.902413

**Published:** 2022-05-18

**Authors:** Seungmin Son, Sang Ryeol Park

**Affiliations:** National Institute of Agricultural Sciences, Rural Development Administration, Jeonju, South Korea

**Keywords:** CRISPR/Cas9, new breeding technology, plant organelle, recalcitrant elite crop, transgene-free genome editing, virus-induced genome editing

## Abstract

The development of plant varieties with desired traits is imperative to ensure future food security. The revolution of genome editing technologies based on the clustered regularly interspaced short palindromic repeats (CRISPR)/CRISPR-associated nuclease 9 (Cas9) system has ushered in a new era in plant breeding. Cas9 and the single-guide RNA (sgRNA) form an effective targeting complex on a locus or loci of interest, enabling genome editing in all plants with high accuracy and efficiency. Therefore, CRISPR/Cas9 can save both time and labor relative to what is typically associated with traditional breeding methods. However, despite improvements in gene editing, several challenges remain that limit the application of CRISPR/Cas9-based genome editing in plants. Here, we focus on four issues relevant to plant genome editing: (1) plant organelle genome editing; (2) transgene-free genome editing; (3) virus-induced genome editing; and (4) editing of recalcitrant elite crop inbred lines. This review provides an up-to-date summary on the state of CRISPR/Cas9-mediated genome editing in plants that will push this technique forward.

## Introduction

The development of new crop varieties has greatly contributed to increasing crop yield; the implementation of new plant breeding technologies is no exception and is now emerging as an appealing solution to overcome the food security crisis caused by climate change and population growth. A classic and traditional plant breeding method involves cross-pollination between genotypes with traits of interest to combine them into one plant; however, crossing brings together two genomes when only a few loci are desired, which necessitates many more generations of backcrossing over long time periods (over 10 years) before obtaining a new crop variety with improved traits such as yield, quality, and greater tolerance to biotic/abiotic stresses (Lusser et al., 2012). Specifically, accelerated elite crop improvement was virtually impossible with these methods (Lenaerts et al., 2019). However, the discovery of genome editing mediated by clustered regularly interspaced short palindromic repeats (CRISPR)/CRISPR-associated nuclease 9 (Cas9) introduced a possible solution to these limitations (Lander, 2016; Stella and Montoya, 2016).

The era of genome editing with nucleases began with zinc-finger nucleases (ZFNs) in 1996, when they were first reported to function as site-specific nucleases. ZFNs are chimeric proteins comprising several zinc finger DNA-binding domains and the non-sequence-specific endonuclease domain of the restriction endonuclease *FokI* (Kim et al., 1996). The target specificity comes from the DNA-binding domain of ZFNs, which is composed of four to six tandem zinc fingers that each recognize a sequence of about 3-base pairs (bp). Since the intact *FokI* endonuclease is a dimer, the ZFN-based system requires two ZFNs, each binding on either side of their target sequence (Petolino, 2015). Although the applicability of ZFNs in genome editing has been validated in both animals and plants, their use is challenging due to their low efficiency, complex construction of the zinc finger region, and severe off-target effects. Notably, the next iteration of sequence-specific nucleases such as transcription activator-like effector nucleases (TALENs) and Cas9 offered a simpler construct design and higher efficiency compared to ZFNs (Nemudryi et al., 2014). Like ZFNs, TALENs comprise the *FokI* endonuclease domain, but their DNA-binding specificity is conferred by transcription activator-like effector (TALE) DNA-binding domains, which were identified in the *Xanthomonas* genus causing bacterial disease in crop plants (Jankele and Svoboda, 2014). The DNA-binding domain of TALEs is composed of a generally conserved 33–35 repetitive amino acid motif with high variability at positions 12 and 13 (the variable di-residue or RVD; Boch et al., 2009; Moscou and Bogdanove, 2009). Since each RVD recognizes a 1-bp sequence, in contrast to the 3-bp motif recognized by zinc fingers, sequence specificity can be more precisely engineered in TALENs compared to ZFNs. However, the difficulty in protein engineering TALENs has been a major obstacle to widespread adoption for genome editing (Adli, 2018).

The CRISPR/Cas9 system is based on RNA-guided DNA cleavage to perform genome editing and is highly efficient, providing an alternative to previous genome editing methods relying on protein-guided sequence-specific DNA recognition and cleavage ZFNs and TALENs (Gaj et al., 2013; Li et al., 2020). Current CRISPR systems can be classified into two classes that are further subdivided into six types and 19 subtypes (Shmakov et al., 2017). The CRISPR Class 2 system requires a single Cas protein, whereas the Class 1 system uses a multi-subunit Cas complex (Tang, 2019). Therefore, Class 2 systems have been widely adopted as a means to achieving genome editing. Indeed, the CRISPR/Cas9 Class 2 type II system using a single Cas protein from *Streptococcus pyogenes* (SpCas9) is by far the most studied and used (Pennisi, 2013; Marraffini, 2016). In its original setting in *S. pyogenes*, Cas9 is an endonuclease with RuvC and HNH nuclease domains; cleavage specificity is provided by a CRISPR RNA (crRNA) that is transcribed from a CRISPR array harboring short fragments of foreign DNA molecules encountered by the bacterium. This CRISPR array is processed into small crRNAs that guide Cas9 to the target sequence (in the native case, foreign DNA), resulting in Cas9-directed cleavage of both non-target and target DNA strands, within the crRNA-target DNA complex (Jinek et al., 2012; Nishimasu et al., 2014; Sternberg et al., 2015). In this process, the trans-activating crRNA (tracrRNA), which acts as a bridge between the crRNA and Cas9, is required for crRNA maturation (Deltcheva et al., 2011). Genetic engineering has simplified the *S. pyogenes* CRISPR/Cas9 system down to only two components: Cas9 and a small RNA. A single-stranded single-guide RNA (sgRNA) replaces the crRNA:tracrRNA duplex and contains a unique 20-bp sequence preceding a protospacer adjacent motif (PAM) with the sequence NGG, which is necessary for compatibility with Cas9 (Zhang et al., 2017), while Cas9 was engineered to be targeted to the nucleus. When both Cas9 and the sgRNA are present in the cell, the sgRNA binds to its complementary target site on genomic DNA, allowing the co-complexed Cas9 protein to precisely cleave the site, leading to a double-stranded DNA break (DSB; Jiang and Doudna, 2017). This Cas9-mediated sequence-specific DSB is then repaired by non-homologous end joining (NHEJ) and homology-directed repair (HDR). NHEJ results in insertions or deletions (Indels) of various lengths that often introduce a frameshift in the target coding sequence (Song et al., 2021). NHEJ-mediated knockouts offer a highly precise and efficient method to inactivate genes of interest, making the CRISPR/Cas9 system ideally suited for plant breeding (Marraffini, 2016). HDR is used for gene replacement, protein tagging, and gene stacking, which can be used for scientific research and agriculture (Malzahn et al., 2017).

CRISPR/Cas9 has made remarkable contributions to the plant science research and plant breeding over the last decade. However, major limitations remain that need to be overcome. We provide here a summary of current obstacles related to plant organelles, transgene integration, tissue culture, and recalcitrant elite crops.

## Editing of Plant Organellar Genomes

Plant cells contain two subcellular compartments surrounded by a double membrane, mitochondria, and chloroplasts (Rose, 2019). These bioenergetic organelles are generally accepted to have evolved from separate endosymbiotic events with an aerobic prokaryote and a cyanobacterium, respectively (Jensen and Leister, 2014; Roger et al., 2017). They have their own residual genome, referred to here as mitochondrial DNA (mtDNA) and chloroplast DNA (cpDNA), which contain essential genes encoding proteins involved in various processes, including respiration and photosynthesis (Waters and Langdale, 2009; Roger et al., 2017). Plant mitochondria harbor multiple copies of their circular genome, with sizes ranging from 200 kb to 2 Mb, thus much larger and more complex than mammalian mtDNA (approximately 16 kb; Morley and Nielsen, 2017). Plant mitochondria carry about 60–70 genes encoding various components required for respiration (ATP synthase, NADH dehydrogenase, and cytochrome) and its own transcription and translation system (ribosomal RNA, transfer RNA, ribosomal proteins, and RNA polymerase; Small et al., 2020). Moreover, recent studies have revealed that the metabolism of mitochondria integrates many diverse processes in plant cells (Moller et al., 2021). Chloroplasts are an equally important plant organelle for photosynthesis, converting light energy and atmospheric carbon dioxide into oxygen and sugar compounds, and housing the biosynthetic pathways of valuable metabolites (Finkeldey and Gailing, 2013; Nielsen et al., 2016). Each chloroplast also contains several copies of its circular, double-stranded genome, ranging in size from 107 to 218 kb in different plant species and harboring 100–250 genes necessary for photosynthesis and respiration (Rubisco, photosystem, ATP synthase, NADH dehydrogenase, and cytochrome) and, just like mitochondria, its own transcription and translation system (Palmer, 1985; Ramundo and Rochaix, 2015; Daniell et al., 2016). Chloroplasts have long been recognized as a potentially powerful bioreactor to drive high expression of transgenes and high accumulation of foreign proteins due to the high copy number of the chloroplast genome and the ability to shield against gene silencing (Oey et al., 2009). Therefore, editing of the plant mitochondrion and chloroplast genome would clearly empower the study of gene function and offer new ways to improve traits in crops.

To allow the manipulation of plant organellar genomes, several key technologies were developed based on the incorporation of a transgene *via* homologous recombination at a gene of interest for gene functional studies or in a so-called neutral site (Boynton et al., 1988; Svab et al., 1990; Bharadwaj et al., 2019; Rascón-Cruz et al., 2021). Mitochondrial transformation has not been achieved in higher plants (Li et al., 2021a). Although the plant plastid transformation has been successful, there are also limitations associated with its use, such as the frequent inability to obtain progeny harboring the transgene, as well as the need for specialized instruments (Piatek et al., 2018; Arimura et al., 2020). Importantly, organellar genome editing might now be achievable by expressing genes encoding sequence-specific nucleases in the organelles or in the nucleus (Mahapatra et al., 2021; Hanson et al., 2013). The introduction of a transgene into the mitochondrial or chloroplast genome is mediated by homologous recombination and thus relies on DSB formation in the organellar genome (Li et al., 2021a; Mahapatra et al., 2021). Genome editing based on the sequence-specific nucleases ZFNs, TALENs, and CRISPR/Cas9 leads to precise DSBs that may therefore increase the efficiency of genome editing in these plant organelles. Homologous recombination can now be largely circumvented by introducing transgenes encoding sequence-specific nucleases into the nucleus and targeting the protein to the organelles for editing. To allow for their use in the modification of the mitochondrial and chloroplast genomes, sequence-specific nucleases were redesigned by adding a targeting peptide such as a mitochondrial targeting sequence (MTS) or chloroplast transit peptide (CTP) specific to each organelle. Most recently, Kang et al. (2021) reported TALEN plasmids based on bacterial cytidine deaminase (CD) rather than *FokI* or *TevI* for mtDNA/cpDNA editing. They generated double-stranded DNA deaminase (DddA) toxin A-derived cytosine base editor (DdCBE) plasmids targeting mtDNA (mt-DdCBE) and cpDNA (cp-DdCBE) *via* fusion of MTS and CTP, resulting in the efficient introduction of point mutations in the relevant organellar genome, up to 25% in mitochondria and 38% in chloroplast, by mt-DdCBEs and cp-DdCBEs in lettuce (*Lactuca sativa*) and rapeseed, respectively. Although admittedly this would work for ZFNs and TALENs, but not as easily with Cas9.

**Figure 1:**
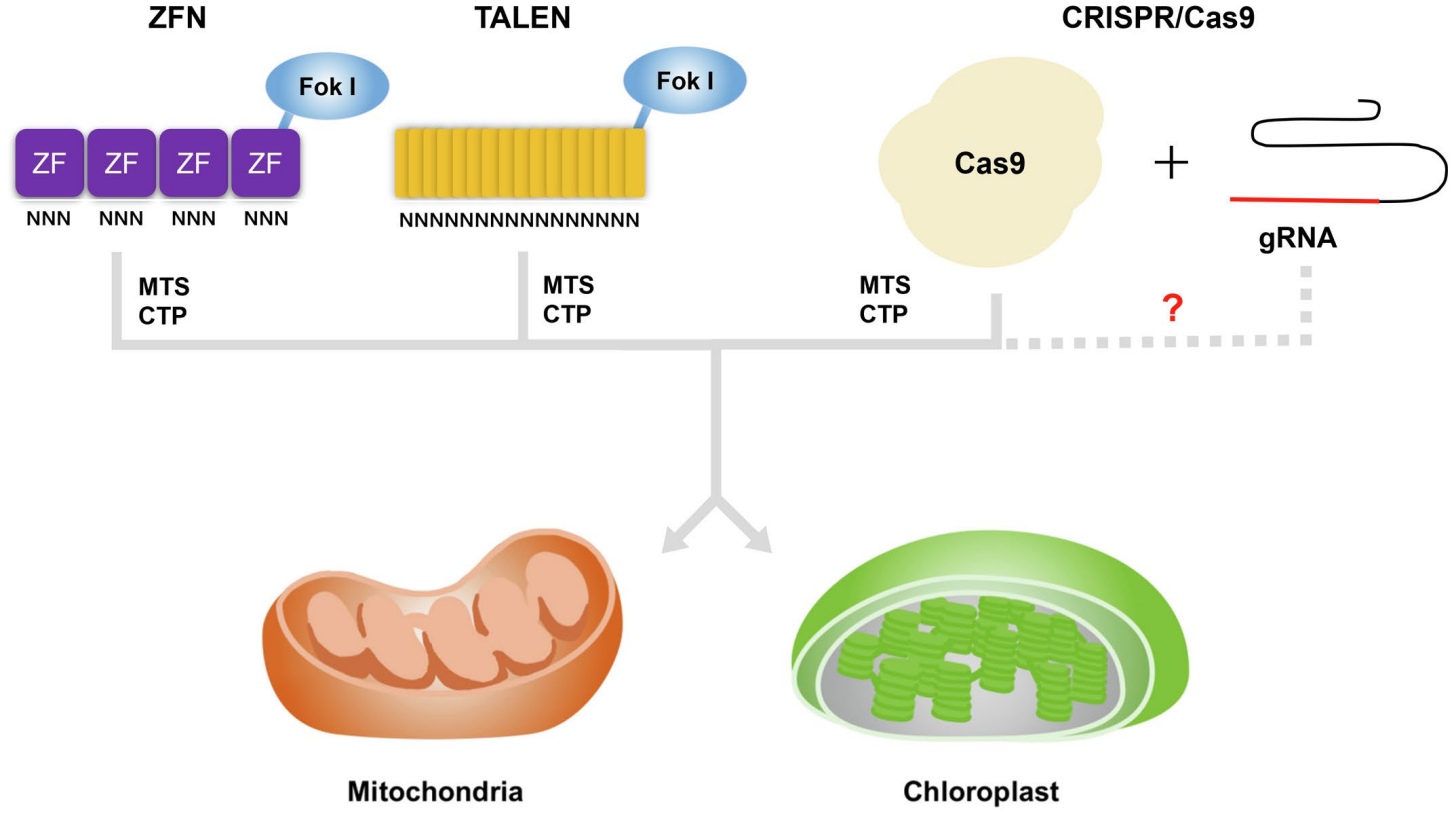
Strategies to import genome editing reagents into mitochondria or chloroplasts in plant cells. Adding a targeting peptide such as a mitochondrial targeting sequence (MTS) or chloroplast transit peptide (CTP) to zinc-finger nucleases (ZFNs), transcription activator-like effector nucleases (TALENs), and clustered regularly interspaced short palindromic repeats-associated nuclease 9 (Cas9) allows their translocation into mitochondria or chloroplasts. In human HEK293K cells, the addition of an RNA transport-derived stem loop element to the sgRNA resulted in its mitochondrial import (Hussain et al., 2021). However, equivalent RNA sequences that might target sgRNAs to mitochondria or chloroplasts are currently unknown in plants.

Unlike ZFNs and TALENs, the CRISPR/Cas9 system uses a specific RNA to recognize the target DNA sequence, making it vastly simpler and more efficient for nuclear genome editing in various crop plants (Li et al., 2021c). Despite its efficiency, the application of CRISPR/Cas9 to the mtDNA and cpDNA faces one major obstacle. Indeed, CRISPR/Cas9-mediated editing requires that both the sgRNA and the Cas9 endonuclease are located in the organelle whose genome is being edited. Notably, sgRNA import into these organelles, which are surrounded by a double-membrane envelope, is highly challenging due to strong electrochemical potential (Glass et al., 2018). Although RNA import of unmodified sgRNAs and mitochondrial genome editing *via* CRISPR/Cas9 has been reported in human cells, the results are controversial (Jo et al., 2015; Gammage et al., 2018; Yang et al., 2021), underscoring the need for methods that facilitate the transfer of sgRNAs into mitochondria/chloroplasts (Figure 1). Recently, two groups have reported the delivery of nucleic acids into mitochondria or chloroplasts using physical transfection and have shown the integration of exogenous DNA into the organellar genomes. Bian et al. (2019) developed the mito-CRISPR/Cas9 system, which relies on a single-stranded DNA (ssDNA) containing a short homologous arm. Delivery of this ssDNA and a specific sgRNA into mitochondria was achieved by microinjection and led to the integration of the ssDNA at the targeted locus in human cells and zebrafish (Bian et al., 2019). Although this report hinted that ssDNA and sgRNA can be imported into mitochondria without modifications, it lacked molecular evidence showing cleavage of the target site by Cas9 (Bian et al., 2019; Tang et al., 2021; Yang et al., 2021). Another group developed a CRISPR/Cas9 system based on so-called edit plasmids that can replicate autonomously and produce Cas9 and the sgRNA while also harboring the donor DNA inside mitochondria or chloroplasts (Yoo et al., 2020). These plasmids are introduced into the Chlamydomonas (*Chlamydomonas reinhardtii*) chloroplast or yeast (*Saccharomyces cerevisiae*) mitochondria by biolistic microprojectile transformation, resulting in the knock-in of donor DNA at the targeted site by homologous recombination (Yoo et al., 2020). Since this CRISPR/Cas9 system allowed genome editing of the mitochondrial or chloroplast genome in distantly related organisms, its optimization for multicellular plants should provide a powerful tool in various crop plants. Another possible avenue of improvement for CRISPR/Cas9 systems is the use of modified sgRNAs targeted to mitochondria by adding the RNA transport-derived stem loop element (RP-loop) to the sgRNA, which allows its import into mitochondria and was shown to result in the knockdown of the transcripts levels of the targeted mtDNA gene in human cells (Hussain et al., 2021). This study suggested that sgRNA can be designed to be imported into organelles by co-opting the native RNA import machinery. However, the mechanisms of RNA import into organelles differ greatly between organisms and are largely unknown in plants (Jeandard et al., 2019; Tian et al., 2020). Nevertheless, several experimental results should be considered for the application of modified sgRNAs to plant mtDNA/cpDNA editing. For instance, voltage-dependent anion channels (VDACs) are mainly involved in transfer RNA (tRNA) import into plant mitochondria (Salinas et al., 2006, 2014; Michaud et al., 2014). For chloroplasts, adding an internal 110-bp sequence derived from the eggplant latent viroid to the 5′ end of the green fluorescent protein (GFP) mRNA resulted in the import of this chimeric RNA into the chloroplasts of *Nicotiana benthamiana* (Gómez and Pallás, 2010a,b). In addition, the single-stranded RNA genome of bamboo mosaic virus (BaMV) was recognized and imported into *N. benthamiana* chloroplasts by chloroplast phosphoglycerate kinase (chl-PGK; Cheng et al., 2013). The implementation of sgRNA import into plant organelles to edit their genome will therefore entail harnessing the current state of knowledge and better understanding the mechanisms behind RNA import into mitochondria/chloroplasts to yield a simple and efficient solution to edit plant organellar genomes.

## Transgene-Free Genome Editing

**Figure 2:**
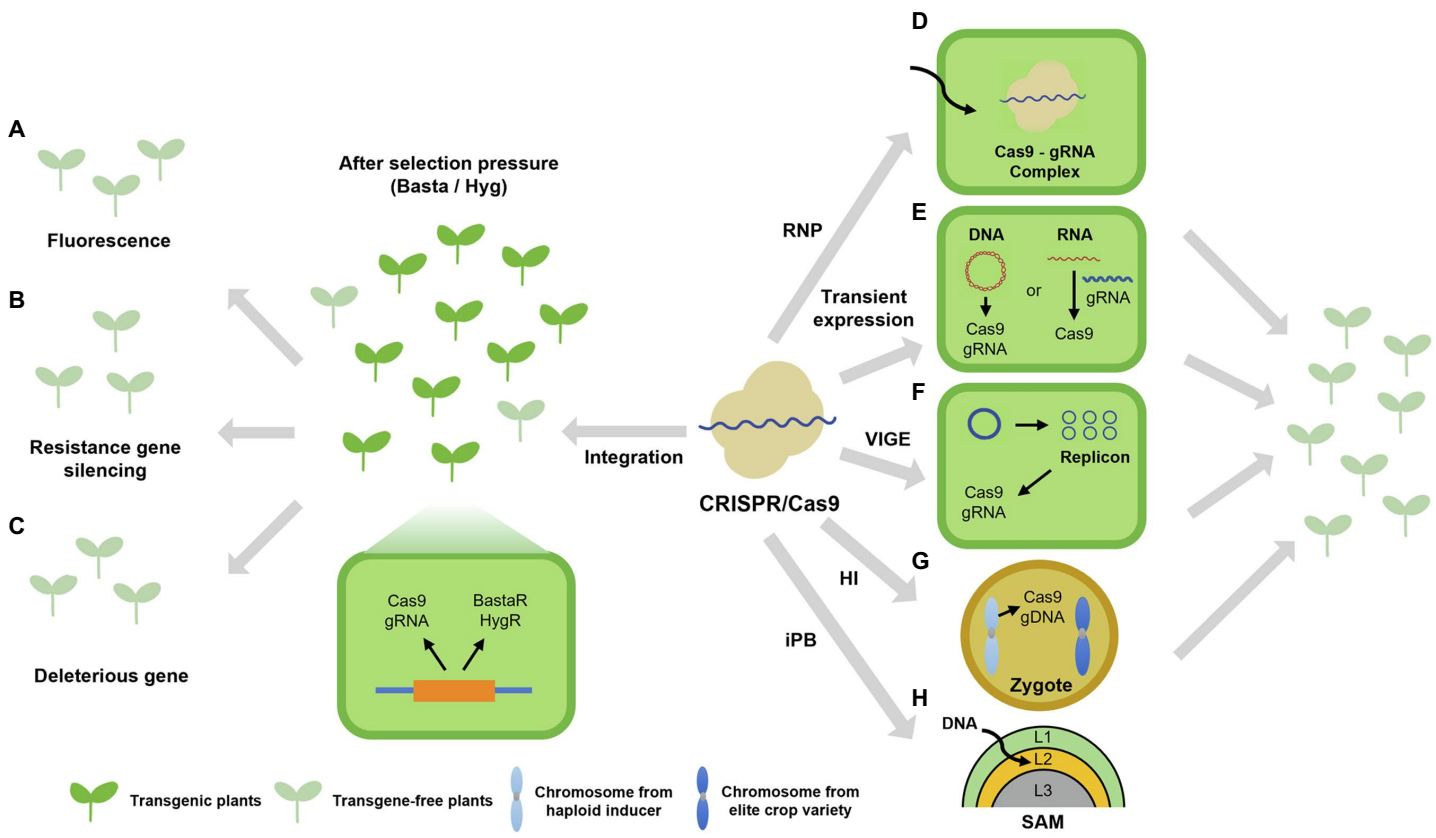
Schematic overview of strategies for the generation and isolation of transgene-free edited plants. **(A–C)** Strategies for the isolation of transgene-free edited plants. The designed constructs encoding the CRISPR/Cas9 system and selection markers, such as fluorescent proteins in seeds (Gao et al., 2016; Stuttmann et al., 2021; **A**), the hairpin RNA interference element silencing *CYP81A6* and conferring sensitivity to the herbicide bentazon (Lu et al., 2017; **B**), and deleterious genes (He et al., 2018; Stuttmann et al., 2021; **C**), were separately introduced into plants by Agrobacterium-mediated transformation. The constructs integrate into the plant genome and express both *Cas9* and the selection markers. After identification of edited plants, plants lacking the trait conferred by the selection marker can be isolated as transgene-free efficiently. **(D–F)** Strategies for the generation of edited plants without the stable integration of a transgene. Several CRISPR/Cas9 systems allow gene editing without requiring the stable integration of a transgene. Preassembly of a ribonucleoprotein containing purified Cas9 and *in vitro* transcribed sgRNA (Woo et al., 2015; Malnoy et al., 2016; Subburaj et al., 2016; Svitashev et al., 2016; Liang et al., 2017; Andersson et al., 2018; Toda et al., 2019; Nicolia et al., 2021; **D**); transient expression system based on CRISPR/Cas9 DNA or RNA (Zhang et al., 2016; Chen et al., 2018; **E**); and viral vectors designed for CRISPR/Cas9 (Oh et al., 2021; **F**). **(G)** Transgene-free genome editing based on haploid induction. Crossing a haploid inducer line carrying the CRISPR/Cas9 transgene (*Cas9* and sgRNA) with the wild-type (WT) plant produces zygotes with one chromosome derived from the haploid inducer line and one chromosome derived from the WT. Since the chromosomes from the haploid inducer line are later eliminated, so is the transgene expressing *Cas9* and the sgRNA, leaving only edited chromosomes derived from the WT; diploid transgene-free edited plants can then be obtained by diploid induction. **(H)** The *in planta* genome editing based on *in planta* particle bombardment (iPB) method. Biolistic delivery of plasmids expressing CRISPR/Cas9 system into the L2 cell layer of shoot apical meristem (SAM) generates stable transgenic wheat lines without callus culture and regeneration (Hamada et al., 2018; Liu et al., 2021).

CRISPR/Cas9-mediated genome editing can introduce small InDels or substitutions at the target site when the need is to inactivate or modify an existing target gene. In most cases of plant genetic engineering, foreign genes are introduced into plants and stably integrated into the plant genome *via* Agrobacterium (*Agrobacterium tumefaciens*)-mediated transformation (Gelvin, 2003). Integration of the CRISPR/Cas9 cassette can lead to undesirable off-target effects, plant lethality, and limitations in conducting functional studies related to specific developmental or physiological processes due to constitutive gene expression (Abdallah et al., 2015; Modrzejewski et al., 2020). Although it could be managed through spatiotemporal gene expression controlled by recombinases and inducible promoters (Wang et al., 2020; Tian and Zhou, 2021), the presence of foreign gene in chromosome raises another important issue such as genetically modified organisms (GMO). After genome editing, the CRISPR/Cas9 and sgRNA construct is no longer needed and can be segregated away; the resulting transgene-free edited crop plants would then be indistinguishable from natural variants (Chilcoat et al., 2017). In fact, genome-edited crops are not considered GMO in several countries and are thus cultivated without the typical restrictions associated with GMOs (Turnbull et al., 2021). For this reason, transgene-free genome-edited plants are mainly obtained through laborious and time-consuming genetic segregation, which can be especially challenging for crops with large polyploid genomes. Therefore, various technologies are being developed and implemented to identify transgene-free plants more efficiently (Figure 2).

Transgene-free edited plants can be accomplished through two distinct methods: (1) segregating the transgene out after its stable integration and (2) bypassing stable integration entirely and relying on a transient system. The first issue at stake here can be reformulated as how to select transgene-free edited plants in the first place, as their proportion in the entire pool is low, with individual plants traditionally identified *via* PCR with transgene-specific primers. As a workaround, simplified screening methods have been designed based on fluorescence (Gao et al., 2016; Stuttmann et al., 2021), gene silencing (Lu et al., 2017), or deleterious genes (He et al., 2018; Stuttmann et al., 2021). But why spend so much time trying to remove the transgene? Is it possible to edit the plant genome without integration of a transgene? Since Agrobacterium-mediated transformation culminates with the stable integration of the T-DNA, other delivery and/or expression methods are needed. In mammalian systems, genome editing has been successfully achieved *via* the delivery of preassembled ribonucleoproteins (RNPs) consisting of purified recombinant Cas9 and *in vitro* transcribed sgRNA molecules (Kim et al., 2014; Liang et al., 2015). An advantage of this approach also lies in reduced changes for off-target mutagenesis, as the RNP is rapidly degraded once inside the cell (Vakulskas and Behlke, 2019). Notably, the delivery of RNPs into plant cells is complicated by the physical barrier imposed by the cell wall. However, the cell wall can be digested away when isolating protoplasts, followed by transfection with the polyethylene glycol (PEG) method. Accordingly, genome-edited plants have been regenerated from protoplasts transfected with *in vitro* preassembled CRISPR/Cas9 RNPs in Arabidopsis, tobacco, rice, lettuce, and potato (*Solanum tuberosum*) without the need for a selectable marker due to high mutagenesis frequencies (Woo et al., 2015; Andersson et al., 2018). Although regeneration of genome-edited plants was not reported, RNP-mediated genome editing in protoplasts was also achieved in wheat (*Triticum aestivum*), petunia (*Petunia hybrida*), grapevine (*Vitis vinifera*), apple (*Malus domestica*), and tomato (*Solanum lycopersicum;* Malnoy et al., 2016; Subburaj et al., 2016; Liang et al., 2017; Andersson et al., 2018; Nicolia et al., 2021). Another transient transfection approach focuses on the introduction of CRISPR/Cas9 RNPs in rice zygotes (Toda et al., 2019). Indeed, since the cell wall forms after gamete fusion, the RNP is transferred during *in vitro* fertilization of gametes; the subsequent regeneration of genome-edited rice plants reached a frequency of 14–64% in positive editing events. Biolistics provides another efficient means for the delivery of RNPs in plants, as demonstrated in embryos from maize (*Zea mays*) and wheat, achieving gene editing (Svitashev et al., 2016; Liang et al., 2017). It should be noted that setting up the RNP system might be difficult for many laboratories due to technical challenges inherent to the method and the lack of selection pressure.

Aside from RNPs, many gene expression systems have been described for transgene-free genome editing in plants that can be divided into DNA-dependent and DNA-free methods. For instance, the transiently expressing CRISPR/Cas9 DNA (TECCDNA) system is based on a plasmid expressing *Cas9* and the sgRNA, and obtained transgene-free genome-edited plants and delivered into plant cells *via* biolistics; TECCDNA applied to wheat calli reached a success rate of up to about 5% (Zhang et al., 2016). An Agrobacterium-mediated transient expression system was also devised for targeted mutagenesis. In a proof-of-concept, the tobacco (*N. tabacum*) *phytoene desaturase* (*PDS*) gene was selected as a target, whose loss of function is easily observed in *albino* plants. The resulting CRISPR/Cas9 binary plasmid harboring the T-DNA, named hCas9-NtPDS, was introduced into Agrobacterium cells, which were then used to infect tobacco leaf discs (Chen et al., 2018). At least 8.2% of the regenerated *albino* plants were transgene-free. The use of Agrobacterium and T-DNA constructs has the potential for integration, which can be difficult to confirm for small degraded plasmid fragments. DNA-free transient expression methods would avert these issues entirely. Rather than introducing a DNA plasmid encoding the needed components for CRISPR/Cas9-mediated editing, transcripts for Cas9 and the sgRNA can be produced *in vitro* and transiently transfected into cells. Hence, the TECCRNA system (by analogy with TECCDNA) was introduced into wheat callus by biolistic bombardment and achieved some level of genome editing, albeit with a low mutagenesis rate of about 1% (Zhang et al., 2016). The difficult of *in vitro* transcription and the low editing efficiency are the constraints of TECCRNA system. Addressing these limitations requires new approaches, whereby the components needed for editing may be expressed from a viral system. Other techniques such as virus-induced genome editing (VIGE), the haploid induction (HI)-mediated method, and *in planta* particle bombardment (iPB) method may also generate transgene-free edited plants; these methods are discussed below.

## Virus-Induced Plant Genome Editing

Most genome-edited crop plants are regenerated from callus or protoplasts by tissue culture. However, plant cell transformation and regeneration methods can vary greatly from plant to plant and need to be optimized each time (Altpeter et al., 2016). In addition, tissue culture is also demanding, costly, time-consuming, and laborious, presenting a serious bottleneck for crop plants (Espinosa-Leal et al., 2018). Although the expression of embryogenic or morphogenic regulators can improve the transformation efficiency of recalcitrant plants and regeneration from protoplasts, this method is still limited in its scope of applications and is labor-intensive (Lowe et al., 2016; Sakamoto et al., 2021). Therefore, producing edited seeds *via* CRISPR/Cas9 without using tissue culture would be invaluable for plant science and agriculture. Virus-induced gene editing (VIGE) solves both the method of transgene delivery and whole-plant regeneration while bypassing tissue culture. Autonomously replicating plant virus-based vectors allows the nucleic acid template to produce a desired protein and RNA rapidly and transiently (Abrahamian et al., 2020). Moreover, the infiltration of plant leaves with a viral vector harboring *Cas9* and the sgRNA often leads to germline mutations when the virus spreads systemically through the plant. Therefore, various plant viral vectors are being developed from viruses with DNA, positive-strand RNA, and negative-strand RNA genomes for the production of Cas9 proteins and/or sgRNA (Oh et al., 2021). Viral vectors nonetheless also have their own sets of limitations related to the delivery capacity of these viral vectors and their systemic delivery to the germline and meristematic tissues.

DNA viral vectors derived from geminiviruses such as bean yellow dwarf virus (BeYDV) and wheat dwarf virus (WDV) can drive the expression of *Cas9* and the sgRNA simultaneously, yielding a high frequency of genome editing in various plants (Baltes et al., 2014; Butler et al., 2015, 2016; Cermak et al., 2015; Gil-Humanes et al., 2017; Wang et al., 2017). However, since geminiviruses are small plant-infecting viruses comprising a single-stranded circular DNA ranging in size from 2.7 to 5.2 kb, packing a recombinant viral genome harboring a large foreign gene into the geminivirus capsid is limited (Brown, 2008). Therefore, early work did not achieve systemic delivery due to the genetic instability and delivery capacity limitations of BeYDV- and WDV-based vectors (Baltes et al., 2014; Butler et al., 2015, 2016; Cermak et al., 2015; Gil-Humanes et al., 2017; Wang et al., 2017). By contrast, negative-strand RNA rhabdoviruses have genomes ranging in size from 11 to 15 kb that can accommodate insertions of up to 6 kb (Jackson and Li, 2016; Burrell et al., 2017). Therefore, a new generation of CRISPR/Cas9 editing vectors was designed based on the two rhabdoviruses barley yellow striate mosaic virus (BYSMV) and sonchus yellow net rhabdovirus (SYNV; Gao et al., 2019; Ma et al., 2020). These engineered rhabdovirus vectors co-expressing *Cas9* and the sgRNA introduced mutations in the target site of the *N. benthamiana* genome with high efficiency. In particular, genome editing of systemic leaves (that is, those not directly infected with the viral vector) was observed in *N. benthamiana* plants infiltrated with the SYNV vector, while editing was restricted to leaves inoculated with BYSMV, with a genome editing frequency of over 90% at the target locus in regenerated plants (Ma et al., 2020). Since most RNA viruses, with the exception of retroviruses, cannot integrate into the host genome (Desfarges and Ciuffi, 2012; Ma et al., 2020), CRISPR/Cas9-mediated genome editing *via* RNA viral vectors results in transgene-free genome-edited plants somatically, but does not produce edited seeds, which is a major drawback.

**Figure 3:**
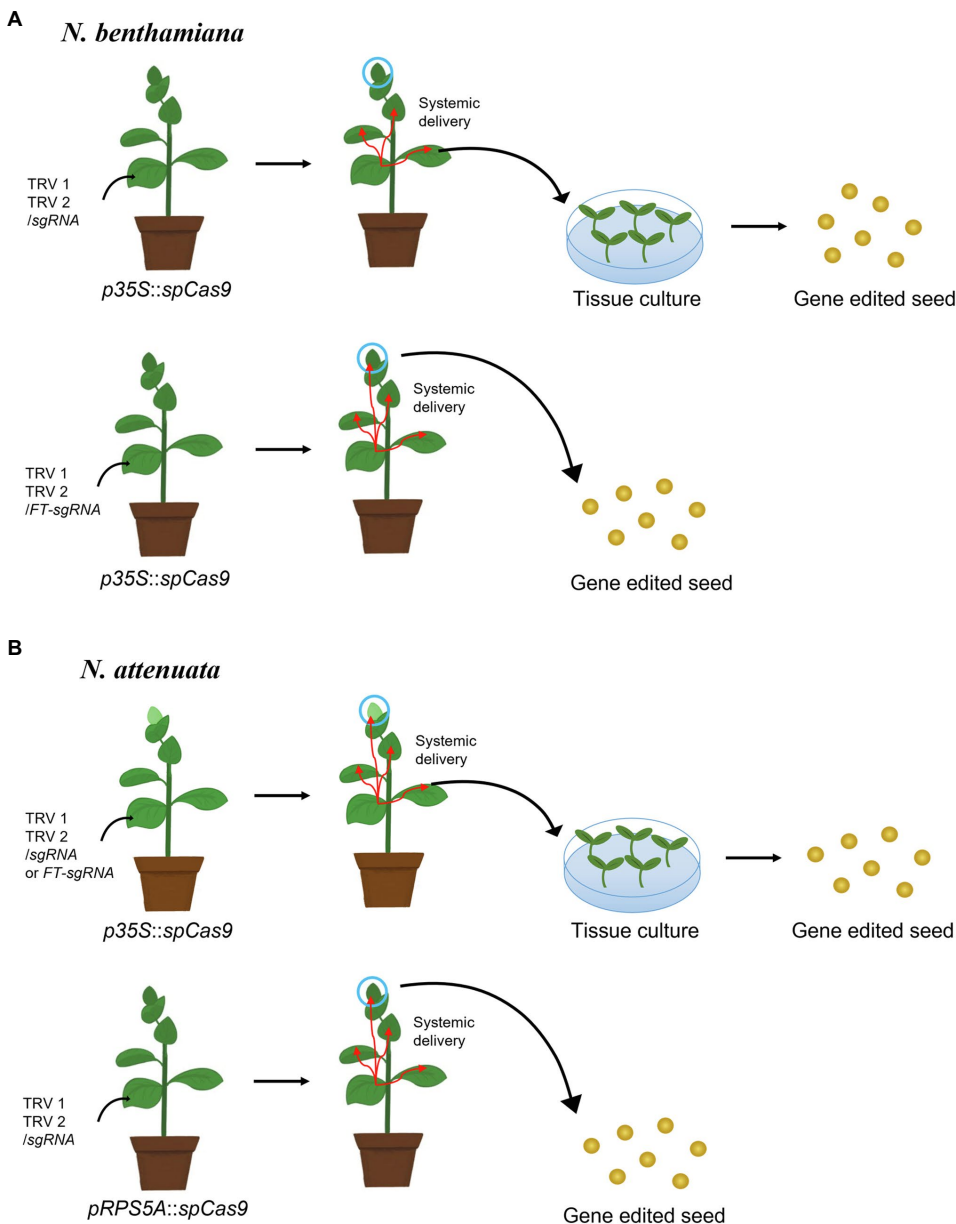
Schematic illustration of tobacco rattle virus (TRV)-mediated seed genome editing in tobacco relatives. **(A)** In *Nicotiana benthamiana*, a TRV construct harboring a sgRNA cloned downstream of the Arabidopsis *flowering locus T* mRNA (TRV2/*FT-*sgRNA) delivers the sgRNA efficiently into the germline, while TRV/sgRNA does not. In addition, the 35S promoter can drive transcription in the germline of *N. benthamiana*. Therefore, infiltration of Agrobacterium cells carrying TRV2/*FT-*sgRNA into *N. benthamiana* leaves expressing *SpCas9* from the 35S promoter results in high-efficiency genome editing in seeds (Ellison et al., 2020). **(B)** In *Nicotiana attenuata*, infiltration of TRV/sgRNA or TRV2/*FT-*sgRNA into *N. attenuata* plants expressing *SpCas9* from the 35S promoter does not lead to genome editing in seeds, as the 35S promoter is insufficient to induce the germline mutation in *N. attenuata*. However, the RPS5A promoter can induce high gene expression levels in the germline, resulting in genome editing in seeds when leaves are infiltrated with TRV2/sgRNA into *N. attenuata* plants expressing RPS5A-driven *SpCas9* (Oh and Kim, 2021).

Other viruses can reach the germline; indeed, viral vector-based gene editing in the germline was first reported with tobacco rattle virus (TRV). TRV is a member of the *Tobravirus* genus, whose genomes consist of bipartite positive-strand RNAs and can infect over 400 plant species. Its wide range of hosts, together with systemic virus movement to all plant tissues including the germline and meristematic tissue, suggested that TRV might be a useful viral vector for genome engineering (Bradamante et al., 2021). Indeed, the transient expression of the TRV-based vector in jasmine tobacco (*N. alata*) delivered meganuclease to the *dihydroflavonol 4-reductase* (*DFR*) gene and introduced mutations at the desired target site in reproductive organs such as pollen grains with low frequency (Honig et al., 2015). Although meganuclease coding sequences are small (~1 kb) and thus work well with the packing limitations imposed by viral capsids, engineering them to specifically recognize desired target sites is extremely difficult (Iqbal et al., 2020). Therefore, several groups developed a CRISPR/Cas9-based TRV genome editing system and succeeded in obtaining mutated seeds. Because the delivery capacity of TRV is less than 3 kb, the Cas9 expression cassette was not included into the vectors, opting instead for transgenic plants constitutively expressing *SpCas9* (Figure 3). In one approach, Ali and coworkers co-inoculated the TRV1 and TRV2 vectors expressing the sgRNA under the control of the pea early browning virus (PEBV) promoter into the leaves of transgenic *N. benthamiana* plants expressing 35S-driven *SpCas9*, which resulted in the introduction of germline mutations, as confirmed by the screening of progeny seed, although the efficiency was low and only observed for seeds produced from early flowers (Ali et al., 2015). In fact, the frequency of heritable genome editing was less than 0.23% and was attributed to inefficient sgRNA movement to the germline. One solution to increase sgRNA movement entailed fusing the sgRNA to the mRNA of Arabidopsis *flowering locus T* (*FT*), which can migrate from the leaf vascular tissue to the shoot apical meristem. Inoculating the leaves of transgenic *N. benthamiana* plants expressing *SpCas9* from the 35S promoter (Ellison et al., 2020) with the modified TRV1 vector resulted in dramatically improved heritable mutagenesis, even up to 100%. The effectiveness of the *FT* mRNA in raising the frequency of heritable genome editing was independently confirmed with a VIGE system based on cotton leaf crumple virus (CLCrV) in Arabidopsis plants overexpressing *SpCas9* (Lei et al., 2021). Surprisingly, in contrast to *N. benthamiana* and Arabidopsis, *SpCas9* expressed from the 35S promoter does not lead to mutations in the germline of coyote tobacco (*N. attenuata*), even when the sgRNA is fused to the *FT* mRNA (Oh and Kim, 2021), likely due to low expression from the 35S promoter in this species and specific tissue (Wilkinson et al., 1997; Sunilkumar et al., 2002; Patro et al., 2012). However, transgenic *N. attenuata* plants expressing *SpCas9* from the ribosomal protein S5 A (RPS5A) promoter induced high levels of expression in all plant tissues, including the germline and meristematic tissues; inoculation of leaves with TRV vectors carrying the sgRNA successfully obtained genome-edited seeds (Oh and Kim, 2021).

Viral vectors other than TRV have also been reported to introduce heritable genome editing events, with viral vectors such as CLCrV in Arabidopsis and barley stripe mosaic virus (BSMV) in wheat (Lei et al., 2021; Li et al., 2021b). Importantly, these other viral vectors still rely on transgenic plants expressing *SpCas9* and have not been modified to express *SpCas9* directly, such that delivery capacity remains a primary issue. A possible solution may be within reach with the hypercompact Cas9 named CasΦ, almost half the size of SpCas9, which was discovered in huge bacteriophages and shown to function in human cells and plant cells (Pausch et al., 2020). Therefore, we anticipate that the incorporation of smaller *Cas9* sequences into the viral vectors together with the sgRNA as well as an efficient delivery method of the germline and meristematic cells will resolve many of the bottlenecks associated with the VIGE system.

## Genome Editing of Recalcitrant Elite Crop Inbred Lines

While CRISPR/Cas9-mediated genome editing provides a new avenue for new breeding technology (NBT) and crop trait improvement, most elite crop varieties and inbred lines used in commercial breeding are recalcitrant to common transformation methods. For example, Agrobacterium- and virus-mediated transformation methods are ineffective in many crop varieties lacking the necessary host susceptibility factors (Hwang et al., 2017; McLeish et al., 2019). Biolistic bombardment and regeneration from protoplasts are also challenging in many elite crop cultivars (Kelliher et al., 2019; Ren et al., 2020; Reed and Bargmann, 2021). Since these recalcitrant elite cultivars cannot be directly transformed with the relevant constructs, one proposed method calls upon crossing elite cultivars with other varieties that are more amenable to transformation with the constructs needed for CRISPR/Cas9-mediated genome editing, in sum using one plant as a delivery vehicle.

The transgenic ZC01 maize line expresses *Cas9* and the sgRNA, thus forming an *in vivo* desired-target mutator (DTM) that allowed genome editing at target loci in non-transformable elite maize lines upon crossing (Li et al., 2017). A similar concept was implemented in 2019 with a hybrid gene modification method named haploid inducer-edit (HI-EDIT) and haploid-inducer-mediated genome editing (IMGE), which combine the two NBT technologies CRISPR/Cas9-mediated genome editing and haploid induction (HI; Kelliher et al., 2019; Wang et al., 2019). The constructs expressing *Cas9* and the sgRNA are introduced into transformable maize inbred lines; the resulting lines are then crossed to a haploid inducer line harboring a mutation in *matrilineal* (*MATL*)/*not like dad* (*NLD*)/*phospholipase A1* (*PLA1*), which results in pollen with defects in male genome transmittance and, thus, the elimination of the entire paternal genome in some progeny (Kelliher et al., 2017). Transgenic lines harboring Cas9, the sgRNA of interest, and the *matl* mutation are then crossed to recalcitrant elite inbred lines. Fertilization brings Cas9 and the sgRNA in the vicinity of the elite cultivar genome and edits the desired target site, while the *matl* mutation ensures that all paternal chromosomes are removed, leaving only one haploid copy of the elite genome that has been edited. In addition, the male genome carries the transgenes, such that *matl*-mediated haploid induction eliminates the transgenes, thus producing elite cultivar lines that are transgene-free. While the *MATL* locus offers a natural solution for genome reduction, dicot plants such as Arabidopsis lack *MATL* but can also be edited *via* HI-EDIT using an engineered haploid inducer line expressing a variant of *centromeric histone H3 (CENH3;* Kelliher et al., 2019). Notably, the use of *CENH3*-based HI has also been demonstrated in wheat and maize, suggesting its wide potential for application (Lv et al., 2020; Wang et al., 2021). Despite the implementation of the *MATL*- and *CENH3*-mediated HI editing system in several monocot and dicot plants, its widespread use is currently hindered. How HI is regulated and modulated should be explored to increase the overall efficiency of this method. The identification of functional orthologs of *MATL* and *CENH3* in various crops is also challenging. The development of simple and efficient methods improving HI in self-fertilizing plants and chromosomal doubling should be a priority to increase the reach of this method as well.

Recently, commercial wheat varieties have also been mutated successfully *via* iPB method, which delivers gold particles coated with CRISPR/Cas9 system into shoot apical meristem (SAM) of imbibed seeds (Liu et al., 2021). Initially, iPB technology was developed as noble elite wheat transformation method omitting tissue culture and regeneration procedure (Hamada et al., 2017). Since the L2 cell layer of SAM is destined to develop into germ cells such as pollen and egg cells (Carles and Fletcher, 2003), biolistic DNA delivery into the L2 cell layer resulted in generation of stable transgenic wheat lines without callus culture and regeneration (Hamada et al., 2017). Moreover, as a result of applying CRISPR/Cas9 system to iPB technology, in plant genome editing of recalcitrant elite wheat cultivars was achieved (Hamada et al., 2018; Liu et al., 2021). In particular, introduction of transient expression plasmid into L2 cell layer of SAM by iPB method produced the edited elite wheat cultivar without intergradation of transgene (Hamada et al., 2018). However, iPB method based on DNA can cause a small degraded plasmid fragments which have the potential for integration into the chromosomes. Therefore, the development of iPB technologies based on DNA-free genome editing methods will confer the genome editing technical advance for recalcitrant elite crop cultivars without tissue culture, regeneration, antibiotic selection, and intergradation of transgene.

## Conclusion

CRISPR/Cas9 has revolutionized all aspects of plant biology, including functional analysis, mutant library construction, and crop improvement. Utilization of this powerful technology in crop improvement opens a wide avenue to overcoming the threat to food security in the future. Yet, there are still more challenges to overcome. Here, we summarized and discussed some of these issues. Although some progress has been made, important questions remain to be addressed. The discovery of RNA sequences capable of delivering the sgRNA into mitochondria and chloroplasts will unlock genome editing for these organelles. Identification of viral vectors that accommodate large inserts that encode Cas9 and the sgRNA, as well as small Cas enzymes, will be critical to bypassing the issues related to transgene integration and plant tissue culture. In addition, the identification of valuable genes inducing the generation of haploid plants in different crops and the development of iPB technologies based on DNA-free genome editing methods will bring a new era for elite crop improvement.

## Author Contributions

SS and SRP conceptualized. SS wrote the manuscript and designed figure. SRP supervised. All authors contributed to the article and approved the submitted manuscript.

## Funding

This research was funded by Research Program for Agricultural Science and Technology Development (project no. PJ01570601) and supported by the 2022 Fellowship Program (project no. PJ01661001) of the National Institute of Agricultural Sciences, Rural Development Administration, Republic of Korea.

## Conflict of Interest

The authors declare that the research was conducted in the absence of any commercial or financial relationships that could be construed as a potential conflict of interest.

## Publisher’s Note

All claims expressed in this article are solely those of the authors and do not necessarily represent those of their affiliated organizations, or those of the publisher, the editors and the reviewers. Any product that may be evaluated in this article, or claim that may be made by its manufacturer, is not guaranteed or endorsed by the publisher.
